# Outcome Analysis of Palliative Endoscopic Biliary Stenting (EBS) for Frail Elderly Patients Aged 80 Years and Above With Choledocholithiasis

**DOI:** 10.7759/cureus.101378

**Published:** 2026-01-12

**Authors:** Zhi Jing Chua, Shiaw Hooi Ho

**Affiliations:** 1 Internal Medicine, NHS Ayrshire & Arran, Ayr, GBR; 2 Gastroenterology, Hospital Picaso, Selangor, MYS

**Keywords:** cholangitis, choledocholithiasis, ercp, palliative, recurrence

## Abstract

Objectives: This study aims to retrospectively analyse the outcome of palliative endoscopic biliary stenting (EBS) in very elderly patients aged 80 years and above with choledocholithiasis.

Methods: Between September 2017 and January 2022, the endoscopy unit at Universiti Malaya Medical Centre (UMMC) conducted 85 ERCP procedures on patients aged 80 and above, who had been diagnosed with choledocholithiasis. The study sample was narrowed down to 11 patients who had received palliative EBS after excluding cases where stone removal was done and patients undergoing active management. Information regarding the patients’ medical records, including their past medical history, details of the endoscopic retrograde cholangiopancreatography (ERCP) procedure with palliative EBS, and their subsequent follow-up records, was gathered from the UMMC electronic medical record.

Results: Out of the 11 patients included in the study, six patients (54.44%) experienced recurrent cholangitis, with a median interval of 534 days from the date of index ERCP to the onset of the first recurrence. Two of the six patients (33.33%) who experienced recurrence were able to achieve stone clearance after undergoing repeat ERCP. Among the five patients who remained recurrence-free, the median follow-up duration was 120 days. In addition, out of the study cohort, seven patients (63.64%) passed away during the follow-up period, with only one patient (14.29%) dying due to recurrent cholangitis, while the other six patients (85.71%) died of their co-morbidities.

Conclusion: ERCP with palliative EBS can be a feasible alternative for very elderly patients with complex medical issues; however, it is imperative to ensure close follow-up and careful monitoring during the post-treatment period.

## Introduction

Elderly individuals are often associated with frailty, which is an age-related condition characterised by the progressive decline of multiple physiological systems and diminished homeostatic reserve. This decline results in a vulnerable health state in which even minor stressors, such as mild infections or minor procedures, may precipitate disproportionately severe and potentially life-threatening consequences. A previous study demonstrated that between one-quarter and half of individuals aged 85 years and above experience increased susceptibility to falls, disability, long-term care dependency, and mortality [1]. The presence of multiple comorbidities further exacerbates the complexity of frailty, amplifying procedural risks and postoperative complications in this population. Consequently, frail elderly patients often exhibit reduced tolerance for interventions that are otherwise well-tolerated by younger adults. Besides that, according to a statistic from 2015, Malaysia’s life expectancy was 74.87 years, which exceeds the average for several developing countries but remains lower than that observed in more advanced nations [2].

Minimally invasive endoscopic procedures have been the gold standard treatment for choledocholithiasis. Stone removal through endoscopic retrograde cholangiopancreatography (ERCP) is usually the first line of treatment [3]. Larger choledocholithiasis (defined as more than 10mm in size) often requires a longer procedure time and a higher sedation dose to cover such a procedure, especially when stone clearance is contemplated. It also requires the use of specialised equipment and tools and may require the ERCP procedures to be repeated multiple times. All these factors contribute to the increased risk associated with the procedures. As such, for certain frail elderly patients with large choledocholithiasis, a standard ERCP with sphincterotomy and biliary stenting without further attempt to remove the stone may be carried out in order to reduce procedure-related risk. Following index ERCP where stone clearance is not achieved, there are generally two approaches: the radical or conservative approach [4,5]. For the radical approach, the patient will be scheduled for repeated ERCP to achieve complete stone clearance or direct surgery (cholecystectomy & common bile duct (CBD) exploration), while the conservative approach means that repeated ERCP is not scheduled until biochemical/clinical biliary obstruction occurs. The radical approach may not be suitable for elderly patients with poorer physical conditions, and thus, a conservative approach, which is the palliative endoscopic biliary stenting (EBS), can sometimes be the preferred option for frail elderly patients with high surgical risks.

EBS is associated with a promising survival rate in high-risk elderly patients who have not achieved complete stone removal [6]. Some studies have also demonstrated that EBS can help to decrease the size and number of stones, as well as facilitate stone removal for a period of time [7,8]. While the efficacy of biliary stenting in the short term has been demonstrated, its potential advantages in the long term are not well-established. The European Society of Gastrointestinal Endoscopy (ESGE) is strongly against the use of long-term EBS due to a high complication rate of 34-63% (mainly cholangitis) and a high mortality rate of 2.3-23.5% on a medium-term follow-up [4]. Along with that, the American Society of Gastrointestinal Endoscopy (ASGE) guidelines indicate that management should target periodic stent replacement, with no suggestions for a permanent solution through EBS [3]. Stent-related complications, such as stent migration or occlusion, tend to happen after a period of time after stent implantation, which could lead to recurrence of biliary obstruction and acute cholangitis [5]. These highlight the importance of determining the suitable candidates for the procedure and only utilise palliative EBS on elderly patients with co-morbidities and a limited life expectancy. The ESGE guidelines quoted the 2-fold risk of procedure-related death in individuals older than 80 years of age and suggested that palliative EBS should be approached with caution [4].

Justifications and aims

Although several guidelines have recommended stent replacement every 3-6 months, it can be challenging for very elderly patients with multiple health conditions to comply with the suggestions of undergoing further ERCP procedures. Moreover, while ERCP is considered a safe procedure, it carries a certain number of risks. ERCP comes with a mortality rate of 0.5% to 2%, and up to 5% chance of morbidity (in the increasing order of frequency), such as perforation, bleeding, cholangitis, and pancreatitis. In addition to this, therapeutic ERCPs have a 7-fold chance of having serious complications compared to diagnostic ERCPs [9]. As a result, patient selection plays a significant role, especially in high-risk elderly patients.

Besides that, biliary obstruction caused by choledocholithiasis behaves differently from biliary strictures. The stent patency tends to be longer than the conventional three-month period as biliary drainage does not rely solely on the patency of the stent in the former. Instead, biliary drainage in choledocholithiasis occurs both within and around the stent. The behaviour of biliary obstruction caused by choledocholithiasis can vary due to the different causes of obstruction. Strictures in the bile duct can cause complete blockage of the duct, while stones may permit some bile flow even if they are causing blockage. Another explanation for this observation is that the stent is able to keep the stones apart, preventing them from causing total blockage. Therefore, biliary drainage can still take place around the stent even though there may be a complete stent occlusion [10]. Hence, it is imperative for physicians to be aware of these differences in order to provide the most appropriate and effective treatment for their patients.

Without clear guidelines regarding the use of palliative EBS and lack of evidence in the very elderly population, there is variation in different practices, and the long-term outcomes of this approach are uncertain. Thus, the effectiveness and suitability of palliative EBS in very elderly patients should be assessed and evaluated again currently. Therefore, this research aims to retrospectively study the outcome of palliative EBS in the very elderly patients aged 80 years and above with choledocholithiasis. 

The understanding of the duration of symptom recurrence pattern in the local population would greatly help the physicians to provide better advice and guidance to the patients and their families during the counselling for palliative EBS. This information will also enable the physicians to closely monitor the patients, especially towards the time when the symptoms are expected to recur. This level of monitoring and attention can help to ensure that any further treatments or interventions that are necessary can be performed in a timely manner, leading to a more positive and desirable outcome for the patients.

## Materials and methods

Study design

**Figure 1:**
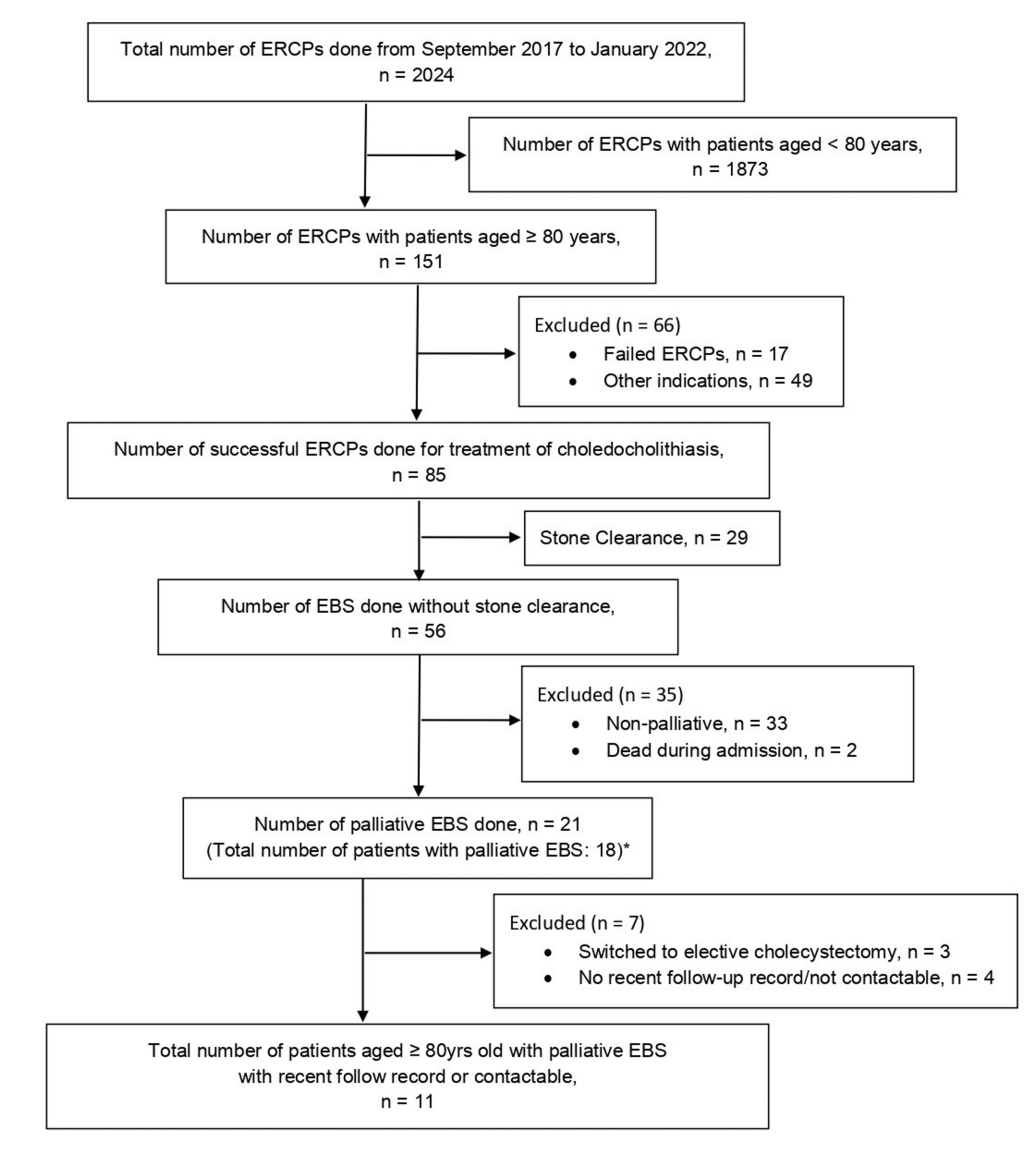
Patient Flow Chart ERCP: Endoscopic Retrograde Cholangiopancreatography; EBS: Endoscopic Biliary Stenting *Three patients had recurrence and underwent repeat palliative EBS during this time period.

The present study was conducted at Universiti Malaya Medical Centre (UMMC), Malaysia and was approved by the institutional review board of UMMC Medical Research Ethics Committee (MREC). Data for the ERCP cases that were performed from the initiation of the electronic endoscopy reporting system in September 2017 till January 2022 were collected from the UMMC electronic medical record (EMR) and analysed. Only frail elderly patients who were aged 80 years and above with choledocholithiasis, who failed to achieve stone clearance following the index ERCP and were subjected to palliative EBS without further attempt to remove the stone, were eligible to be included in this study. Patients who passed away on the same admission during the index ERCP and those who switched from palliative care to planned ERCP or elective cholecystectomy will be excluded from this study. No routine or scheduled ERCP was carried out for any of the patients included in the study. Patients were followed up at the hepato-pancreato-biliary (HPB) or gastroenterology clinic either monthly to every six months, depending on the patient’s condition. During the follow-up, patients will be reviewed for any recurrence of cholangitis symptoms, such as jaundice, fever and abdominal pain, and blood samples will be taken to assess patient’s liver function tests (LFTs). The patient flow chart in this study is shown in Figure 1. From 1st September 2017 till 31st January 2022, there were 2024 cases of ERCP done at the UMMC endoscopy unit. Among these cases, 1873 were excluded as the patients were younger than 80 years old. Seventeen cases of failed ERCP, which resulted from unsuccessful cannulation or patients being uncooperative, and 49 ERCP cases that were performed for the management of other indications, such as biliary strictures, periampullary tumour and cholangiocarcinoma, were excluded from the study. A total of 85 ERCP cases were done to treat choledocholithiasis, with 29 cases achieving complete stone removal, and 56 cases were done with EBS without stone clearance. Out of the 56 cases of EBS, 33 of them were under active management and two passed away on the same admission during the index ERCP. This resulted in a total of 21 palliative EBS being performed during the specified time period. During the same time frame, of the 21 palliative EBS cases, three patients had recurrence and underwent repeat palliative EBS, resulting in a total of 18 patients receiving palliative EBS. Three patients switched from palliative EBS to elective cholecystectomy, while four patients had no recent follow-up and were uncontactable. In total, 11 patients aged 80 years and above who underwent palliative EBS for the management of choledocholithiasis with recent follow-up or contactable were included in the present study.

A retrospective review of the 11 patients’ medical records was conducted via the UMMC EMR. This involved reviewing patients’ past medical history, as well as records pertaining to the ERCP procedure with palliative EBS, and the subsequent follow-up, to identify any cases of recurrent biliary obstruction or cholangitis. Patients who were unable to complete their scheduled follow-up visits were contacted via phone to gather as much information as possible. Two sets of data were collected: the baseline characteristics of the patients and the clinical outcomes of the palliative EBS. For the baseline characteristics, the following information was collected from each patient: age, race, gender, co-morbidities, antiplatelet use, number and size of stones, as well as the diameter of the stent used. This study examined a range of comorbidities, which might have an impact on the management of patients, including but not limited to pre-existing conditions such as coronary artery disease (CAD), stroke, diabetes mellitus (DM), dementia, and underlying cholelithiasis. The data gathered on clinical outcomes include the presence and cause of cholangitis recurrence, the time duration until recurrence, any achievement of stone clearance after repeated ERCP, the length of follow-up in recurrence-free patients, the mortality rate and the cause of mortality.

ERCP procedure

In general, prior to the ERCP procedure, each patient and/or their families were given information about the procedure, and consent was obtained. The patients will be given suppository Voltaren 100mg shortly before the procedure, except those with a history of allergy to non-steroidal anti-inflammatory drugs. This group of patients with choledocholithiasis and biliary obstruction will be given prophylactic intravenous antibiotics (third-gene cephalosporin) before the procedure as well.

During the ERCP procedure, the patients were placed in a prone position. Whenever available, sedation cover was provided by the anaesthetic team. Deep sedation was achieved through infusion of intravenous propofol. When the anaesthetic support is not available, moderate sedation is normally achieved through bolus doses of intravenous midazolam and fentanyl. Sedation was used to help alleviate patients’ discomfort or anxiety that they might experience during the procedure, ensuring patients’ comfort and relaxation, in order to facilitate a successful outcome. Additionally, during the ERCP, patients’ vital signs were closely monitored through electrocardiogram, blood pressure and pulse rate monitoring, oxygen saturation and carbon dioxide level monitoring. Patients will be given supplemental oxygen through a nasal cannula. The aim of such close continuous monitoring was to ensure the patients’ safety and well-being throughout the procedure.

The ERCP procedure was performed using a side-viewing duodenoscope aided by fluoroscopy support. Bile duct cannulation was achieved with wire-guided cannulation and contrast medium injection methods using a sphincterotome device. Following the injection of the contrast material into the bile duct, the size of the stones can be estimated by using the scope’s diameter on the X-ray film as a reference. In cases where multiple stones were present, the largest stone’s diameter was measured. The EBS procedure generally used plastic stents, of either straight or pigtail shape, and the diameter of stents used was usually either 7 French (Fr) or 10 Fr, while the length of stents varied depending on the features of the choledocholithiasis. After the procedure, all patients and/or their families were given both written and verbal instructions regarding further management. Patients were scheduled for regular follow-up at either the HPB or gastroenterology clinic to monitor for any signs of biliary obstruction or recurrent cholangitis.

Outcomes and definitions

The primary outcome of this study was to analyse the time to symptom recurrence after the index ERCP with palliative EBS in patients aged 80 years and above with choledocholithiasis who failed to achieve stone clearance after the index ERCP. The secondary outcomes were the duration of follow-up for patients who did not develop recurrence, the mortality rate and the cause of mortality. The causes of mortality were categorised into biliary-related and non-biliary-related.

This study considered patients who have had a follow-up within the last six months as recent follow-up cases, and no further actions were taken. In cases where patients who are still alive and have not had follow-up in the past six months, their families were contacted via phone to gather information about their recent well-being and health status. Besides that, under circumstances where patients had more than one stent inserted, this study opted to use the stent with the widest diameter. Furthermore, no further information was collected after complete stone clearance was achieved for patients in this study group who developed recurrent cholangitis and underwent repeat ERCP procedures with stone removal. This means the clinical outcomes were only collected and analysed until the point when stone clearance was achieved for this specific group of patients. 

Recurrent cholangitis was defined as the development of cholangitis following the index ERCP, based on the presence of compatible clinical symptoms (including fever, abdominal pain, and jaundice), abnormal biochemical findings (deranged liver function tests and/or raised inflammatory markers), and/or radiological evidence of biliary obstruction on subsequent imaging. Frail elderly patients were operationally defined as individuals aged 80 years and above who underwent palliative EBS during the study period.

Statistical analysis

All data extraction was performed by a single investigator using a predefined data collection template. Simple descriptive statistics were used to analyse both the baseline characteristics of the patients and the clinical outcomes of palliative EBS. Categorical data were described as frequencies (percentages), and continuous variables with normal distribution are expressed as arithmetic mean and standard deviation (SD), while continuous variables with non-normal distribution and discrete variables are presented as median and range. All statistical analyses were performed using Microsoft® Excel® 2019 MSO (Version 2301; Microsoft Corporation, Redmond, WA, USA) [11].

## Results

Baseline characteristics

After excluding patients who did not meet the criteria, a total of 11 patients aged 80 years and above with choledocholithiasis who underwent an ERCP procedure with palliative EBS without stone clearance between September 2017 and January 2022 at the UMMC endoscopy unit were included in this study. Baseline characteristics of these 11 patients are listed in Table 1. The average age of this group of patients was 84.55 ± 3.17 years, and there was no significant difference in terms of gender distribution, with five male patients (45.45%) and six female patients (54.55%). The largest racial group in this study was Chinese, with 9 out of 11 patients (81.82%) identifying themselves as Chinese. One patient (9.09%) identified as Malay, while another patient (9.09%) identified as Indian. The results for the prevalence of comorbidities in this study group were as follows: 3 out of 11 patients (27.27%) had CAD, three patients (27.27%) had a history of stroke, four patients (36.36%) had DM, three patients (27.27%) had dementia, and cholelithiasis was present in nearly half of the study group, with five patients (45.45%) having underlying stones in the gallbladder. In this study group of 11 patients, four patients (36.36%) were found to be using antiplatelet medications, which are commonly prescribed as part of the secondary prevention treatment for stroke and ischaemic heart disease.

**Table 1:** Baseline Characteristics CBD: Common Bile Duct; SD: Standard Deviation; Fr: French (unit)

Parameter	Value
Age, mean (SD), years	84.55 (3.17)
Gender, n (%)	
Male	5 (45.45)
Female	6 (54.55)
Race, n (%)	
Malay	1 (9.09)
Chinese	9 (81.82)
Indian	1 (9.09)
Comorbidities, n (%)	
Coronary Artery Disease	3 (27.27)
Stroke	3 (27.27)
Diabetes Mellitus	4 (36.36)
Dementia	3 (27.27)
Underlying Cholelithiasis	5 (45.45)
Antiplatelet Use, n (%)	4 (36.36)
Number of Stones, n (%)	
Single	5 (45.45)
Multiple	6 (54.55)
Largest Dimension of CBD stones, mean (SD), mm	15.18 (6.23)
Diameter of Stent, n (%)	
7Fr	5 (45.45)
10Fr	6 (54.55)

In terms of the details regarding the palliative EBS procedure, five patients (45.45%) were found to have a single stone, whereas six patients (54.55%) were diagnosed with multiple stones in the CBD. The mean size of the largest dimension of CBD stones measured among the 11 patients in this study was 15.18 ± 6.23 mm. Of the 11 patients, six patients (54.55%) received a 10Fr stent during their ERCP with a palliative EBS procedure, while the other five patients (45.45%) received a 7Fr stent. 

Clinical outcomes

The clinical outcomes of the ERCP with palliative EBS procedure for the 11 patients studied are presented in Table 2. In this study, of the 11 patients, six patients (54.55%) developed recurrent cholangitis during the follow-up period. Among the patients with recurrent cholangitis, four out of the six patients (66.67%) with recurrent cholangitis were found to have stent occlusion, whereas the remaining two patients (33.33%) experienced recurrent cholangitis due to stent migration. The median duration from the date of the ERCP procedure to the onset of the first episode of recurrent cholangitis was 534 days, with a range of 32 to 830 days. Of the six patients with recurrent cholangitis, two patients (33.33%) achieved complete stone clearance following repeat ERCP procedures.

**Table 2:** Clinical Outcomes of ERCP with a Palliative EBS Procedure ERCP: Endoscopic Retrograde Cholangiopancreatography; EBS: Endoscopic Biliary Stenting

Parameter	Value
Recurrent cholangitis, n (%)	6 (54.55)
Causes of cholangitis, n (%)	
Stent migration	2 (33.33)
Stent Occlusion	4 (66.67)
Interval to first recurrent cholangitis, median (range), days	534 (32-830)
Achieved stone clearance after repeated ERCP, n (%)	2 (33.33)
Follow-up duration for those without recurrence, median (range), days	120 (46-1843)
Mortality, n (%)	7 (63.64)
Mortality due to cholangitis, n (%)	1 (14.29)

A noteworthy finding in this study was that out of the five patients who did not develop recurrence, three patients passed away within 2 to 3 months following the index ERCP with palliative EBS, while two patients have remained alive and well to the study date. The follow-up duration for these five recurrence-free patients ranged from 46 to 1843 days, with a median of 120 days. In contrast, the two surviving patients have follow-up durations of 1,857 and 1,892 days, with a median of 1,874.5 days. During the follow-up period, the mortality rate of these 11 patients was 63.64%, with 7 out of them passing away. Out of the seven patients who passed away during the follow-up period, only one patient (14.29%) died due to cholangitis, with the other six patients dying due to various other causes, including hospital-acquired pneumonia, metastatic rectosigmoid cancer, colon cancer, old age, or septic shock resulting from another cause.

## Discussion

According to a study by Meine and Baron, the incidence and risk of specific adverse events that happen during the ERCP procedure vary by age, with a higher prevalence among older patients [12]. Surprisingly, a study found that pancreatitis, which has always been reported as the most frequent post-ERCP complication, was not the most common adverse event observed among elderly patients, and instead, bleeding and cardiopulmonary complications, which could potentially lead to death, were more prevalent [13]. This can be attributed to the fact that elderly patients tend to be associated with underlying health conditions, an increased number of medications, periampullary diverticula, and multiple CBD stones, all of which could make the procedure more complex and increase the risk of post-ERCP complications.

Additionally, very elderly patients may have a greater sensitivity to sedation, which can also contribute to the occurrence of complications. Elderly patients are frequently affected by cardiopulmonary complications related to sedation during the ERCP procedure. Finkelmeier et al. conducted a recent study investigating the effects of sedation during ERCP on the incidence of adverse events in elderly patients and found that advanced age was associated with a higher risk of adverse events during sedation, particularly in very elderly patients [14]. Specifically, these patients are at increased risk of developing complications, such as myocardial infarction, bradycardia, and hypoxaemia during ERCP sedation. Hence, physicians often hesitate to perform repeat ERCP procedures on very elderly patients with complex medical issues if initial stone clearance is not achieved, and instead, they would consider palliative EBS as a viable alternative option.

Interestingly, the current study observed that only a small proportion of ERCP procedures for choledocholithiasis management in very elderly patients utilised palliative EBS. Among the 85 cases of ERCP procedures performed for patients aged 80 years and above with choledocholithiasis, only 21 cases were managed with palliative EBS without stone clearance. Additionally, with three cases switching to active management, only 18 palliative EBS (21.18%) were performed during the study period from September 2017 to January 2022. One possible explanation for the low percentage of palliative EBS performed in elderly patients with choledocholithiasis in this study may be due to patients being managed in this hospital were generally physically healthy and able to tolerate the long procedure for stone extraction or multiple ERCP procedures. Another possibility was that the physicians responsible for these patients were reluctant to use palliative EBS due to the lack of consensus in best practices, as well as the lack of evidence supporting this approach in very elderly patients. Therefore, the results of this study could serve as a reference for physicians in managing elderly patients with choledocholithiasis who are not good candidates for active management or lengthy procedures, which may help to guide physicians in providing more tailored, patient-centred care to this vulnerable population.

Previous studies have shown a biliary stent patency of approximately 3 to 6 months. It has been recommended that the biliary stent should be replaced every 3 to 6 months to prevent stent occlusion, which could result in the recurrence of biliary obstruction or cholangitis [15-17]. In contrast, a previous article suggested that when a biliary stent is used in the management of choledocholithiasis, it can stay in place for a longer period than the duration of the required stent patency. It suggested that the biliary stent does not function as the only conduit for bile flow in the case of CBD stones. It was believed that there remains a lumen in the CBD after the placement of a biliary stent, which allows the flow of bile even if the stent is completely obstructed [10]. A retrospective study was done by Tohda et al. to evaluate the stent-exchange interval in elderly patients with choledocholithiasis. The study compared and analysed the clinical outcomes of the following three groups of patients. Group A underwent regular stent exchanges every six months, Group B had stent exchange scheduled every 12 months, and Group C did not have a regular stent exchange schedule. A conclusion was drawn from the study that exchanging plastic stents at 12-month intervals is a safe procedure for patients with choledocholithiasis [5]. In the present study, the median days from the date of index ERCP with palliative EBS to the first episode of recurrent cholangitis was 534 days, suggesting that the biliary stents employed in the management of choledocholithiasis could last beyond the usual stent patency duration, which is consistent with the above findings.

Besides that, in this study, among the seven patients who passed away during the follow-up period, only one of them died due to recurrent cholangitis, while the remaining six deceased patients died due to their comorbidities, implying that the cholangitis-related mortality is relatively lower than the non-cholangitis-related mortality. Kitagawa et al. also conducted a retrospective study using the propensity score matching to assess and compare the results of palliative EBS and complete duct clearance among elderly patients diagnosed with choledocholithiasis, and their findings also revealed a low percentage of mortality caused by cholangitis in the palliative EBS group, which is in line with the results of this current study [18]. Additionally, in the present study, the median days of the follow-up duration for the five patients who did not experience recurrence were only 120 days, and among them, three patients died within 2 to 3 months after the index ERCP was performed. These findings suggest that most of the patients who underwent the ERCP procedure with palliative EBS were frail elderly patients with multiple medical problems, which might have limited their lifespan. As a result, for very elderly patients with multiple comorbidities and frailty, palliative EBS could be a viable option to alleviate their choledocholithiasis symptoms and improve their overall comfort, especially for those who are unfit and with a limited life expectancy.

The present study observed a moderate rate of recurrence, with slightly more than half of the study group (six patients, 54.55%) experiencing recurrent cholangitis during the follow-up period. All patients who developed recurrence in this study underwent a repeat ERCP procedure, and out of these patients, two patients (33.33%) achieved complete stone clearance after the repeat ERCP. One patient out of the six patients (16.67%) died due to the recurrence, suggesting a relatively low mortality rate from recurrent attacks. This finding indicates that even if patients experience recurrence after receiving palliative EBS, repeat ERCP and re-stenting may be effective in resolving the issue, and in some cases, complete stone clearance might be possible. However, it is important to note that cholangitis can be a serious and potentially life-threatening complication of CBD stones, and a patient’s quality of life can be significantly impacted by its recurrence. The fact that more than 50% of the study population experienced recurrence emphasises the necessity of continuous care and management of this condition. As a result, it is vital to closely monitor and follow up with patients who receive palliative EBS to promptly detect any abnormalities in LFTs or the development of clinical symptoms indicating biliary obstruction or cholangitis, which will allow for timely intervention, such as a change of biliary stents.

Limitations

Several limitations should be considered when interpreting the results of this study. To begin with, the retrospective nature of this study made it impossible to completely eliminate all forms of bias, and the statistical reliability may not be as high as that of a randomised controlled trial. However, conducting a prospective randomised controlled trial in the case of frail and elderly patients can be quite challenging. Second, the limited size of the study sample was a potential issue that needs to be considered. The limited number of participants may not be a full representation of the wider population and may have limited generalisability. The small sample size may also result in a low statistical power of the study, increasing the chances of random variation affecting the results. As a result, this study relied solely on basic descriptive statistics without the application of any comparative statistical analyses. Third, another limitation of this study was that the follow-up of the patients may have been inadequate due to several factors. Firstly, complete tracking of clinical outcomes of all patients was not possible, as the hospitals in Malaysia have their own systems to store patients’ medical records and do not have a unified patient database, which may have resulted in patients undergoing follow-up procedures in other hospitals without their records being available in the UMMC EMR system. Furthermore, some patients with mild recurrence or cholangitis may have opted not to present to the hospital for treatment, which could have led to an underestimation of the true rate of recurrence. In addition to that, it was also possible that spontaneous stone passage may contribute to the lack of recurrence in some of the patients who underwent palliative EBS.

## Conclusions

In summary, this study has shown a moderate rate of recurrence following the index ERCP with palliative EBS and the use of biliary stent in the management of choledocholithiasis was found to be more durable compared to other conditions, such as biliary strictures. In addition, the mortality rate associated with recurrence was low, and most of the recurrent cases were managed effectively, with some achieving stone clearance. It is also worth noting that most of the deceased patients in this study group passed away due to old age or comorbidities, and some died within a short period after the index ERCP.

Therefore, ERCP with palliative EBS can be considered a feasible alternative for high-risk elderly patients who may not be able to tolerate more invasive interventions. Given the advanced age and frailty of such patients, ERCP with palliative EBS may serve as a more suitable and manageable option, despite the limitations of its effectiveness and durability. Hence, given the possibility of recurrence, it is of utmost importance that physicians maintain a vigilant and meticulous approach to post-procedure care following palliative EBS, in order to ensure early detection and management of any recurrence of biliary obstruction, which could lead to complications like cholangitis. Regular follow-up should be scheduled, and patients should be educated on how to identify symptoms suggestive of obstruction or cholangitis and they should promptly contact their healthcare provider if they experience such symptoms. With close monitoring and timely intervention, the risk of morbidity and mortality related to recurrent biliary obstruction and cholangitis can be minimised, thus improving the general well-being and overall quality of life of the very elderly patients. It is also important to note that given the study’s limitations, including potential selection bias and information bias, further research is needed to provide stronger evidence and clearer guidance on the role of palliative EBS in this patient population.
